# Diagnostic model of combined ceRNA and DNA methylation related genes in esophageal carcinoma

**DOI:** 10.7717/peerj.8831

**Published:** 2020-03-31

**Authors:** Xiaojiao Guan, Yao Yao, Guangyao Bao, Yue Wang, Aimeng Zhang, Xinwen Zhong

**Affiliations:** 1 Department of Pathology, Second Affiliated Hospital, China Medical University, Shenyang, China; 2 Department of Thoracic Surgery, First Affiliated Hospital, China Medical University, Shenyang, China; 3 First Affiliated Hospital, China Medical University, Shenyang, China

**Keywords:** MicroRNA, DNA methylation, Esophageal carcinoma, Diagnosis, lncRNA

## Abstract

Esophageal cancer is a common malignant tumor in the world, and the aim of this study was to screen key genes related to the development of esophageal cancer using a variety of bioinformatics analysis tools and analyze their biological functions. The data of esophageal squamous cell carcinoma from the Gene Expression Omnibus (GEO) were selected as the research object, processed and analyzed to screen differentially expressed microRNAs (miRNAs) and differential methylation genes. The competing endogenous RNAs (ceRNAs) interaction network of differentially expressed genes was constructed by bioinformatics tools DAVID, String, and Cytoscape. Biofunctional enrichment analysis was performed using Gene Ontology (GO) and the Kyoto Encyclopedia of Genes and Genomes (KEGG). The expression of the screened genes and the survival of the patients were verified. By analyzing GSE59973 and GSE114110, we found three down-regulated and nine up-regulated miRNAs. The gene expression matrix of GSE120356 was calculated by Pearson correlation coefficient, and the 11696 pairs of ceRNA relation were determined. In the ceRNA network, 643 lncRNAs and 147 mRNAs showed methylation difference. Functional enrichment analysis showed that these differentially expressed genes were mainly concentrated in the FoxO signaling pathway and were involved in the corresponding cascade of calcineurin. By analyzing the clinical data in The Cancer Genome Atlas (TCGA) database, it was found that four lncRNAs had an important impact on the survival and prognosis of esophageal carcinoma patients. QRT-PCR was also conducted to identify the expression of the key lncRNAs (RNF217-AS1, HCP5, ZFPM2-AS1 and HCG22) in ESCC samples. The selected key genes can provide theoretical guidance for further research on the molecular mechanism of esophageal carcinoma and the screening of molecular markers.

## Introduction

Esophageal cancer is a common malignant tumor in the world. It is estimated that there were 572,000 new cases of esophageal cancer and more than 50,000 cases of esophageal cancer deaths in 2018 (Ferlay et al., 2019a). At present, the incidence of esophageal cancer ranks seventh in all tumors, and the mortality rate ranks sixth (Zeng et al., 2016), causing a serious disease burden to humans. Esophageal cancer mainly includes two histological subtypes, esophageal squamous cell carcinoma (ESCC) and esophageal adenocarcinoma (EA) (Xi et al., 2019). Esophageal squamous cell carcinoma is the most common histological type, accounting for approximately 80% of all esophageal cancer cases worldwide (Asombang et al., 2019). The incidence of esophageal squamous cell carcinoma has a distinct geographical distribution, with high incidence in East Asia, East Africa, South Africa, and Southern Europe, and relatively low incidence in North America and other parts of Europe (Ahmed, Ajani & Lee, 2019).

The occurrence of esophageal cancer is the result of a combination of genetic, behavioral, and dietary factors, including family history of esophageal cancer, smoking, drinking, hot drinks, and pickled foods (Andrici & Eslick, 2015; Ohashi et al., 2015; Tai et al., 2017). Despite significant advances in the diagnosis and treatment of esophageal cancer in recent years, patients’ prognosis has not improved significantly, with a 5-year overall survival rate of less than 30% and an even lower survival rate in some developing countries (Hu et al., 1994). The main reason is that early esophageal cancer lacks obvious clinical symptoms, and many patients are already in advanced stage when diagnosed (Codipilly et al., 2018a), resulting in poor prognosis.

At present, the commonly used screening method for esophageal cancer is endoscopy, but it is only implemented in some economically developed areas. Due to the high cost of endoscopy, invasive injury and low acceptability of residents, it is not widely used in many areas, so it has a broad prospect to find sensitive and specific biomarkers for early screening of esophageal cancer (Arantes & Espinoza-Ríos, 2018). It is of great value to reduce the mortality of esophageal cancer and improve the prognosis of patients in epidemic areas and high-risk groups.

With the rapid development of bimolecular technology, the genetic research of esophageal cancer is increasing. A series of susceptible polymorphic sites and tumor related somatic mutations have been found gradually, and the understanding of the genomic characteristics of esophageal cancer is gradually in-depth (Wang et al., 2020; Kalikawe et al., 2019; Yuan et al., 2019). In addition to genetic characteristics, scientists have also found that esophageal cancer has a variety of epigenetic changes in recent years (Lin, Wang & Koeffler, 2018b). Epigenetics is a kind of hereditable genomic changes, which does not include changes in DNA nucleotide sequences. These changes may persist throughout the cell division period, including DNA methylation, histone modification and non-coding RNA (ncRNA). RNA epigenetic changes play a key role in maintaining normal physiological functions and tumorigenesis and epigenetic abnormalities have become a common feature of almost all human tumors (Gherardini et al., 2016). Epigenetic abnormalities not only drive the occurrence and development of tumors, but also affect cell growth, invasion, metastasis, heterogeneity and drug resistance (Pfister & Ashworth, 2017).

More importantly, epigenetic changes are often early events in the process of disease occurrence, which provides an opportunity to find tumor specific biomarkers. In this study, we used the miRNA, mRNA, DNA methylation and clinical data in database to search for potential biomarkers of ESCC by using the knowledge and methods of bioinformatics from the mechanism of ceRNAs. This study will definitely provide theoretical guidance on the molecular mechanism of esophageal carcinoma and the screening of molecular markers.

## Material and Methods

### Data source

The data analyzed in this study involved miRNA, mRNA, DNA methylation and clinical data, all of which were derived from the GEO (Barrett et al., 2007) database at NCBI (https://www.ncbi.nlm.nih.gov/geo/) and humans Cancer Genome Database TCGA (https://www.cancer.gov/) (Akbani et al., 2014). Table 1 listed the specific GEO data numbers used in this study (Li et al., 2014).

**Table 1:** Specific distribution of ESCC data sources.

	GEO/TCGA	Tumor	Normal
miRNA	GSE59973	3	3
	GSE114110	30	10
RNA	GSE120356	5	5
DNA methylation	GSE52826	4	8
Clinical data	TCGA-ESCC	81	

### Screening of differentially expressed miRNAs

The R package (Affy, version 1.52.0) was used for background expression value correction and data normalization of the original data in each dataset (Yuen, Zhu & Leung, 2018). The probes in each file were then annotated according to the appropriate platform annotation file. Probes without matching genetic symbols were removed. When different probes map to the same gene, the average value of all the probes mapped to the gene was the final expression value of the gene. Through literature research and analysis, the miRNA datasets of ESCC in this study were GSE59973 and GSE114110 (Wen et al., 2018). Differential expression analysis was performed and the differential miRNA selection should meet *p* value < 0.05 and log2—FC—>1.

### Differential methylation analysis

In order to confirm the results, we also downloaded the methylation (platform: Illumina HumanMethylation450 BeadChip) and expression (IlluminaHiSeq) microarray data from TCGA database for validation. Through DNA research and analysis, the DNA methylation dataset in this study was determined to be GSE52826 (Li et al., 2014), and the DNA methylation difference analysis was performed with GEO2R (Cao et al., 2019a). The adjusted *P* value less than 0.05, and delta expression value either greater than 1 (up-regulated gene) or less than −1 (down-regulated gene) were as the cut-off value of the expression chip data.

### Target prediction

The 12 miRNAs selected in the above steps were used for target prediction, and the target prediction software RNA22 was used (Jabbar et al., 2019; Wang et al., 2019), which is target prediction software for predicting microRNA binding sites based on sequence characteristics and can be a good theory of ceRNA hypothesis mechanism.

### Hypergeometric test

Hypergeometric testing is the most commonly used prediction method in the ceRNA mechanism (Tay, Rinn & Pandolfi, 2014). After the hypergeometric test calculation (Doi, Takahashi & Kawasaki, 2017), the result *p* value less than 0.05 is the potential ceRNA pair. The specific formula is as follows:

\begin{eqnarray*}P=1-F(x/N,K,M)=1-\sum _{t=0}^{x-1} \frac{ \left( \frac{K}{t} \right) \left( \frac{N-K}{M-t} \right) }{ \left( \frac{N}{M} \right) } \end{eqnarray*}

### Pearson correlation coefficient calculation

The gene expression matrix of GSE120356 was used to calculate the Pearson correlation coefficient (Ma et al., 2019c). Based on the ceRNA mechanism, the two RNAs of the ceRNA pair have a co-expression effect, which can be well supported for subsequent analysis. Therefore, Pearson correlation was taken here and 0.7 had a highly credible co-expression trend (Li et al., 2019a; Li et al., 2019b). Finally, the ceRNA that meets the hypergeometric test threshold was crossed with the ceRNA under the Pearson threshold to obtain the final ceRNA pairs.

### Construction of ceRNA network

The selected differential mRNA, lncRNA, and differential miRNA were paired in the miRcode database. Target gene prediction was performed on the selected differential miRNAs using the starBase (Han et al., 2020) online software (http://starbase.sysu.edu.cn/), and the target genes were predicted by miRDB (Rigden & Fernández, 2020), miRTarBase (Liu, Huang & Luo, 2020), and TargetScan (Dong et al., 2020) databases, thereby obtaining ceRNA regulation of lncRNA-miRNA-mRNA network, using Cytoscape v3.5.1 software (Qi et al., 2019) for mapping.

### Functional and pathway enrichment analyses

Gene Ontology (GO) is a comprehensive information tool providing gene function of individual genomic products, including three aspects: molecular function (MF), biological process (BP) and cellular component (CC). The Kyoto Encyclopedia of Genes and Genomes (KEGG) is a database resource for advanced biological functions and utilities. Both analyses and annotations are based on the DAVID database (https://DAVID.ncifcrf.gov/) (Kumar et al., 2019) and are used to explore and understand the biological significance of specific gene lists. In this study, GO and KEGG analysis of DEGs were based on standard error discovery rate (FDR) <0.05.

### Identification of prognostic lncRNAs from ceRNA network

With the survival time of all esophageal cancers in the TCGA database, the Survival package in the R language was used to perform survival analysis on the differential lncRNA (Shi et al., 2020b). The difference was statistically significant with *P* ≤ 0.05.

### Samples collection and ethics statement

In this study, we collected 10 pairs of ESCC specimens from patients in the First Affiliated Hospital of China Medical University from 2015 to 2018. All patients were confirmed histologically. All patients participating in the study signed the informed consent form and the study was approved by the Institutional Ethics Committee of China Medical University.

### RNA extraction and reverse transcription- quantitative polymerase chain reaction analysis

Total RNA of tissues was extracted, and the sample was fully ground with liquid nitrogen, added 1ml Trizol solution, mixed, and placed at room temperature for 5 min to fully crack. Then, 14,000 g centrifuged at 4 °C for 15 min, and the RNA was divided into three layers, in the upper water phase, and transferred to another new RNase free EP tube. Later, isopropanol was added, gently and thoroughly mixed, and allowed to stand at room temperature for 10 min. At 4 °C, the samples were centrifuged at 14,000 g for 10 min, RNA precipitation was collected and washed twice with 75% ethanol. According to the amount of precipitation, an appropriate amount of DEPC water was added to dissolve the precipitation.

RNA samples were reverse transcribed into cDNA using TransScript One Step gDNA Removal and cDNA Synthesis SuperMix. RT qPCR was performed using TB Green Premix Ex Taq. The PCR conditions included an initial step at 95 °C for 10 min, followed by 40 cycles of amplification and quantification (95 °C for 15 s, 60 °C for 1 min, and 60 °C for 1 min). GAPDH was used as an endogenous control for normalization. The sequences of the primers used for RT qPCR were as follows:

HCG22 forward, 5′ CCTGGGGAGAGGTGTCATTT 3′  and reverse, 5′ TGGTCTCTGGGTGCTTAGTG 3′; RNF217-AS1 forward, 5′  TGGGAATGACAGCAGAAAG 3′  and reverse, 5′  TCCGCAGAGTGAACAAGAA 3′; HCP5 forward, 5′  GATGACTATGGGGTGAGGGG 3′  and reverse, 5′  TATGGAGATGAGGTGTGCCG 3′; BBOX1-AS1 forward, 5′  GGCACATTTGGAAGTT 3′  and reverse, 5′ TCAGGGTAACCGTAGC 3′.

## Results

### Analysis of differentially expressed miRNAs

**Figure 1:**
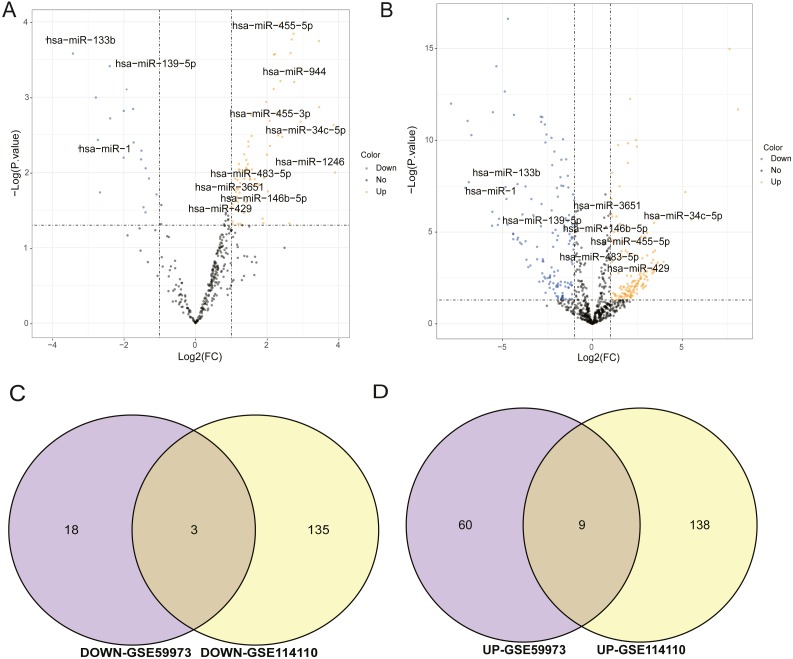
Analysis of differentially expressed miRNAs. (A) Volcanic map of differential miRNA distribution of GSE59973. (B) Volcanic map of differential miRNA distribution of GSE114110. (C) Venn diagram of down regulated miRNA by GSE59973 and GSE114110. (D) Venn diagram of upregulated miRNA by GSE59973 and GSE114110. The numbers in parentheses represent the percentage of the corresponding portion of the total difference.

The miRNA datasets used in this study were GSE59973 and GSE114110, respectively. The threshold of selecting differential miRNAs was *p*.value < 0.05 and log2—FC—>1. In the volcano maps of differential miRNAs of GSE59973 and GSE114110, the black regions in the graph represented the distribution of non-differentiated miRNAs, the red fractions showed significantly differentially up-regulated miRNAs, and the green fractions represented significantly differentially down-regulated miRNAs (Figs. 1A, 1B). At the same time, the differentially up-regulated and down-regulated miRNAs obtained from the two datasets were separately taken for intersection. Finally, three differentially expressed down-regulated miRNAs were obtained, including hsa-mir- 139-5p, hsa-mir-1 and hsa-mir-133b. There were nine up-regulated miRNAs, including hsa-mir-1246, hsa-mir-34c-5p, hsa-mir-455-5p, hsa-mir-455-3p, hsa-mir-146b-5p, hsa-mir-3651 and hsa-mir-429, as shown in Figs. 1C and 1D. These 12 important miRNA differences will be used for subsequent target prediction and ceRNA correlation analysis.

### CeRNA network construction

**Figure 2:**
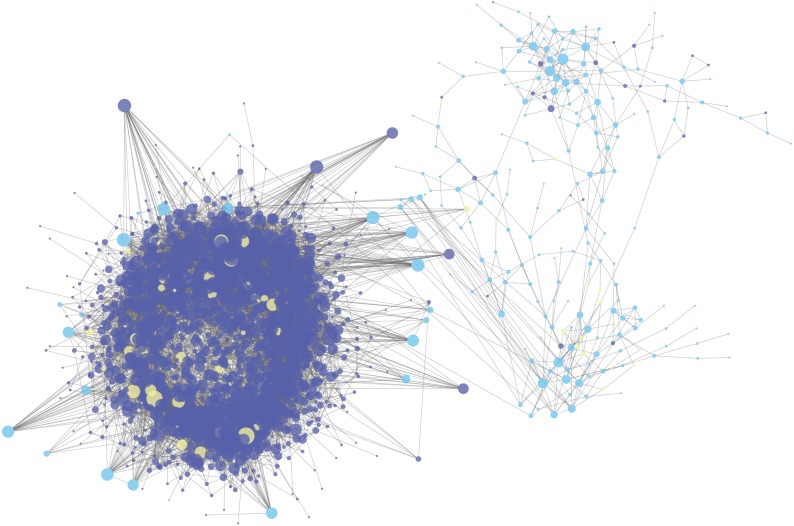
The lncRNA-miRNA-mRNA ceRNA network in ESCC. The deep blue dots represent lncRNA, the light blue dots represent mRNA, and the yellow dots represent miRNA. The size of the dots indicates the degree of node degrees.

The gene expression matrix of GSE120356 was calculated by Pearson correlation coefficient. The ceRNA pair with *r* value more than 0.7 and the ceRNA pair with hypergeometric test value less than 0.05 were selected to determine the final ceRNA relationship for a total of 11,696 pairs. It can be seen that there were complex ceRNAs in ESCC and the network, as shown in Fig. 2, was the ceRNA network constructed in this study. The red dots represent lncRNA, the green dots represent mRNA, and the blue dots represent other types of RNA. The size of the dots indicates the degree of node degrees. It can be seen that the ceRNA network constructed in this study has a clear trend of ceRNA regulation between lncRNA.

### Analysis of ceRNA combined with DNA methylation

**Figure 3:**
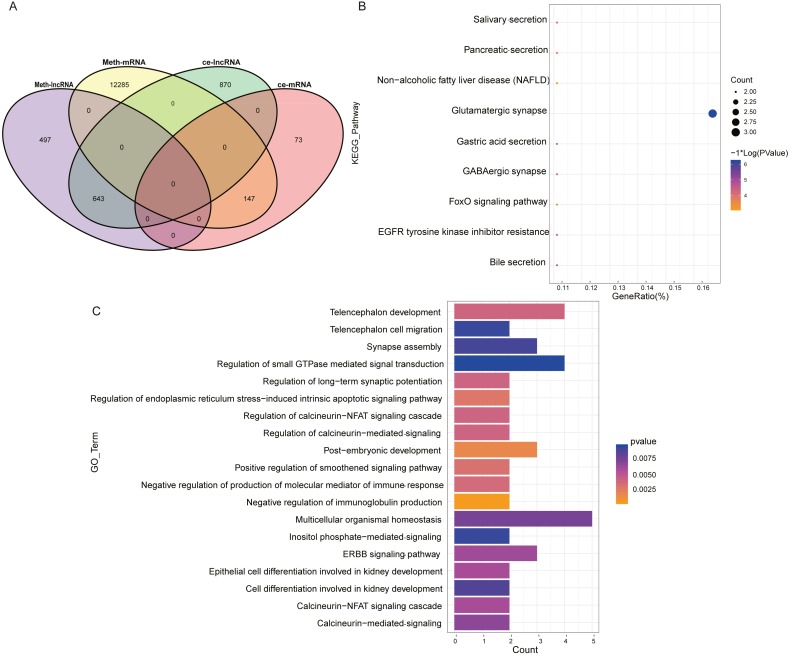
Analysis of ceRNA combined with DNA methylation. (A) Venn diagram of ceRNA and differential methylation associated with lncRNA and mRNA in ESCC. (B) KEGG pathway maps associated with lncRNA in the ESCC ceRNA network. (C) GO-BP pathway maps related to lncRNA in ESCC ceRNA network. The numbers in parentheses represent the percentage of the corresponding portion of the total difference.

The mRNA and lncRNA in the ceRNA network were combined with DNA methylation analysis to find ceRNAs with differential DNA methylation in ESCC. Figure 3A showed the intersection of ceRNA and differentially methylated genes and it could be seen that in the ceRNA network, there were 643 lncRNAs with methylation differences and 147 mRNAs with methylation differences. At the same time, we extracted 64 of the 147 mRNAs that form a ceRNA pair with lncRNA, such as KCNA3, USP44, OPLAH, SMTN, TTC6, COL27A1, SYNE2, LHX1, NRG1 and XKR4. These 64 mRNAs were subjected to GO-BP and KEGG pathway enrichment analysis to discover the biological regulation processes in which these lncRNAs were mainly involved in the ceRNA network. Figures 3B and 3C showed the KEGG pathway bubble diagram and the GO-BP pathway strip diagram of the 64 ceRNAs, respectively. The GO-BP pathway was only visualized by selecting the pathway with pvalue <0.01. It can be seen that for the KEGG pathway results, these significantly enriched pathways were mainly concentrated in the FoxO signaling pathway and related pathways such as glutamate synapses and EGFR tyrosine kinases, and these pathways have been reported to play an important role in ESCC (Zhang et al., 2017; Kashyap & Abdel-Rahman, 2018; Hong et al., 2015). For the bar graph of GO-BP, these mRNAs were mainly involved in the biological processes of the corresponding cascade of calcineurin. The above analysis indirectly indicates that lncRNA in the ESCC ceRNA network indirectly regulates the biological activities and life processes of ESCC.

### Prognosis related lncRNAs

**Figure 4:**
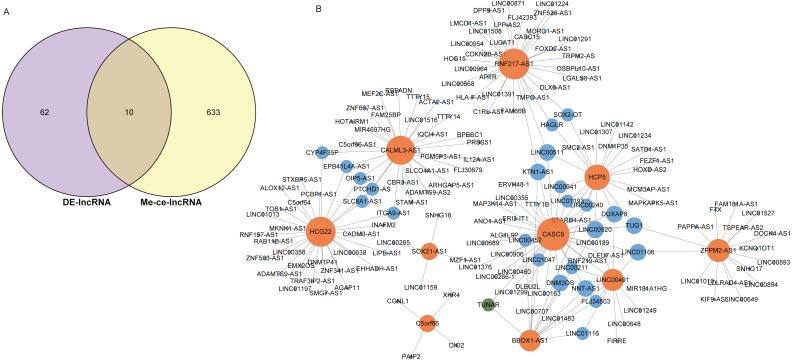
Identification of 10 important lncRNAs. (A) Venn diagrams of 643 methylated lncRNAs and differentially expressed lncRNAs in the ceRNA network. (B) The ceRNA network diagram of 10 important lncRNAs involved in regulation. The numbers in parentheses represent the percentage of the corresponding portion of the total difference.

The intersection of 643 lncRNAs in the ESCC ceRNA network and the differentially expressed lncRNAs in GSE120356 screened 10 important lncRNAs, namely CASC9, SOX21-AS1, HCP5, HCG22, RNF217-AS1, CALML3-AS1, LINC00491, BBOX1-AS1, C5orf66, ZFPM2-AS1, shown in Fig. 4A. To explore the ceRNA regulatory network in which these 10 lncRNAs were involved in ESCC, a subset of the ceRNA networks from which 10 important lncRNAs were extracted, shown in Fig. 4B. The orange dots in the figure indicate these 10 important lncRNAs, the green dots represent mRNAs, and the blue dots represent important lncRNAs other than these 10 important lncRNAs. It can be seen that these 10 important lncRNAs are mainly related to other lncRNAs in this study. There is a clear trend of ceRNA regulation between ncRNA-interacting lncRNAs.

**Figure 5:**
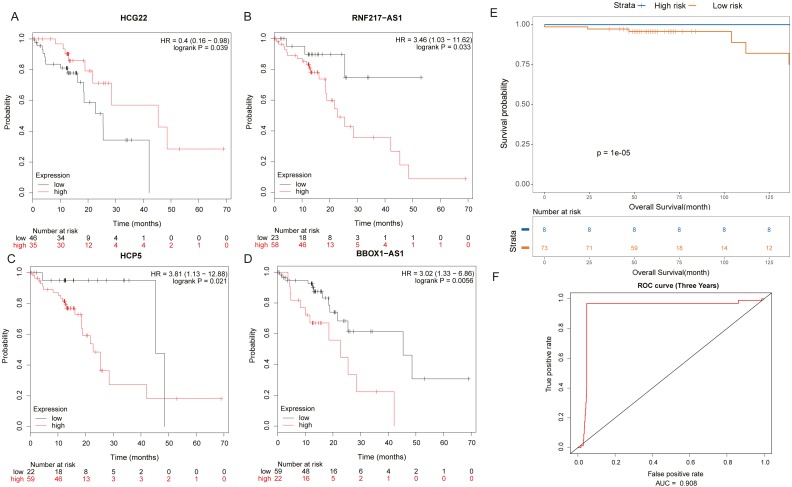
Prognosis related lncRNAs. (A) HCG22, (B) RNF217-AS1, (C) HCP5, (D) BBOX1-AS1 survival curves. (E) Four lncRNA cox survival analysis curves. (F) Four lncRNA prediction model ROC curves.

It was found by analysis that four lncRNAs in these lncRNAs have important effects on the survival prognosis of ESCC. The four lncRNAs were HCG22, RNF217-AS1, HCP5, BBOX1-AS1, and the survival curves were shown in shown in Figs. 5A–5D. At the same time, the survival model was analyzed by ROC curve, and the survival analysis of the next three years was predicted, in which the AUC value reached 0.908 (Figs. 5E, 5F).

### Validation of differential lncRNAs by RT-qPCR

**Figure 6:**
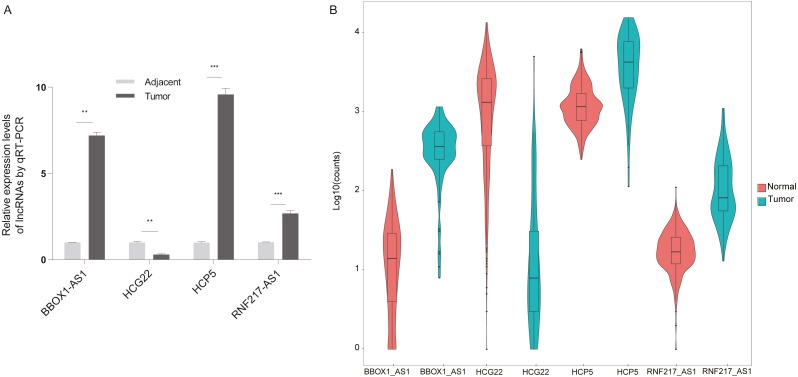
Validation of differential lncRNAs by RT-qPCR. (A) HCG22, RNF217-AS1, HCP5, BBOX1-AS1 validation with qPCR. ^∗∗∗^*P* < 0.001 vs. normal. qPCR, quantitative polymerase chain reaction. (B) Four lncRNA relative expression level in TCGA.

The present study aimed to determine whether the differentially expressed lncRNAs identified in the microarray analysis were up-regulated or down-regulated in clinical esophageal cancer patients. Esophageal cancer specimens and non tumor epithelial tissues were obtained, and the differential lncRNAs were validated with RT-qPCR (Fig. 6A). The experimental results showed that RNF217-AS1, HCP5 and ZFPM2-AS1 expression was significantly up-regulated in ESCC samples compared with healthy tissues, while HCG22 expression was lower than negative controls. These results were consistent with the result of TCGA analysis, as shown in Fig. 6B. Taking together, it was revealed that these differential lncRNAs may be involved in the mechanism of ESCC.

## Discussion

Despite the improvement of clinical diagnosis and treatment in recent years, the accuracy of diagnosis and survival rate of prognosis of esophageal cancer are still low. At present, there is no biomarker with high accuracy for the diagnosis of esophageal squamous cell carcinoma. Therefore, it is of great significance to find biomarkers for the diagnosis of esophageal squamous cell carcinoma from the molecular biological level. In this chapter, we first download microRNA SEQ data from TCGA database by bioinformatics. TCGA is a genetic engineering based on human genome project, which uses sequencing technology for research. The database contains many known and unknown genomes, transcriptome and other information. These data are obtained by the second generation sequencing technology. Second generation sequencing technology is widely used, which can be used in genome single nucleotide polymorphism sequencing (SNP SEQ), methylation sequencing (methel SEQ) and transcriptome sequencing (RNA SEQ). By analyzing the microRNA SEQ data of esophageal cancer from TCGA database, we obtained 12 miRNAs differentially expressed in esophageal cancer and adjacent tissues.

Yang et al. (2013) found that miRNA-338-3p, miRNA-218 and hsa-miRNA-139-5p were up-regulated in esophageal squamous cell carcinoma, while miRNA-183, miRNA-574-5p, miRNA-21 and miRNA-601 were down regulated. Kano et al., (Suzuki et al., 2012) found that 15 miRNAs were down regulated in esophageal squamous cell carcinoma compared with normal tissues, and 4 miRNAs were able to play an anti-cancer role (miRNA-145, miRNA-30a-3p, miRNA-133a and miRNA-133b), which was consistent with the differential expression miRNAs we analyzed, which proved that our screening method was feasible. However, some hsa-mir-1246, hsa-mir-34c-5p, hsa-mir-944, hsa-mir-455-5p and hsa-mir-455-3p have not been reported to be related to the development of esophageal cancer, which is worth further functional study.

Since AFAP1-AS1 has been verified to be differentially expressed in esophageal adenocarcinoma, more and more differentially expressed lncRNAs have been found in tumors. AFAP1-AS1 is a long non-coding RNA amplified from bladder cancer cells by cDNA terminal rapid amplification. It has been confirmed that AFAP1-AS1 was highly expressed in embryonic tissues but not in normal tissues. AFAP1-AS1 was highly expressed in Barrett’s esophagus, non-small cell lung cancer and esophageal cancer (Ji et al., 2018; Wu et al., 2013; Liu, Hu & Wang, 2020a). Gutschner & Diederichs (2012) found that the expression of lncRNA HOTAIR in the primary and metastatic lesions of esophageal cancer was significantly increased, and its expression level in the primary lesions was a powerful predictor of tumor final metastasis and death. Our previous studies have shown that lncRNAs are differentially expressed in esophageal squamous cell carcinoma and matched paracancerous tissues. The abnormal expression of lncRNAs may play an important role in the occurrence and development of esophageal carcinoma. However, the understanding of the whole genome expression pattern and function of lncRNA in esophageal cancer is still limited. In this study, we identified four lncRNAs that play an important role in the survival and prognosis of ESCC, and constructed a multi gene survival prediction model using Cox regression model. At present, it has been found that the above four lncRNAs target genes have been confirmed to participate in the occurrence and development of tumors (Slack & Chinnaiyan, 2019), indicating that the above four lncRNAs are likely to participate in the occurrence and development of esophageal squamous cell carcinoma through their corresponding target mRNA regulation.

The forked head box (Fox) protein family consists of 19 subfamilies of transcription factors, which share a highly conserved DNA binding domain of about 110 amino acids, i.e., the forked head box domain (also known as the winglike helix domain) (Zhang, Virshup & Cheong, 2018; Maiese, 2017; Hou et al., 2018). Forkhead box o (FoxO) transcription factor is considered to be a tumor suppressor that can limit cell proliferation and induce apoptosis (Paik et al., 2007). FoxO gene changes have been described in a limited number of human cancers such as rhabdomyosarcoma (Schöffski et al., 2018), leukemia (Li et al., 2019b) and lymphoma (Liu et al., 2018). In addition, FoxO protein is inactivated by major carcinogenic signals such as phosphatidylinositol-3 kinase pathway and MAP kinase (An et al., 2020). FoxOs regulate the expression of many genes to control many cell functions, such as cell growth (Niimi et al., 2019), survival (Hassell, 2019), metabolism and antioxidant status (Zečić & Braeckman, 2020). However, recent studies have shown new and unknown functions of FoxO in cancer treatment and promotion, which shows that FOXO factor, has complex roles in the disease. EGFR (EGFR, ErbB-1 or HER1) is one of the members of the epidermal growth factor receptor (her) family, which also includes erbB2/HER2, ERBB3/HER3, ErbB4/HER4 (Habban Akhter, Sateesh Madhav & Ahmad, 2018). Her family plays an important role in cell signal transduction, cell proliferation and differentiation. The results of Milas et al. (2004) show that EGFR expression in esophageal squamous cell carcinoma has guiding significance for predicting radiosensitivity. When cells are exposed to high-energy radiation, EGFR outside the nucleus can rapidly enter the cells to form protein complexes without relying on extracellular ligands. It can promote the phosphorylation of DNA protein kinase and repair the break of DNA double strand, thus affecting the radiosensitivity of tumor cells (Gotoh et al., 2007).

DNA methylation can also be used as a biomarker for the diagnosis of esophageal squamous cell carcinoma (Lu et al., 2019). In order to further improve the diagnostic effect of ESCC, we compared the DNA methylation of ESCC and its adjacent tissues on the basis of the above three miRNAs as diagnostic markers. Based on TCGA database and literature search, we analyzed the methylation of KCNA3, USP44, OPLAH and SMTN in esophageal cancer. KCNA3 (potassium voltage-gated channel subfamily a member 3) is a member of the voltage-gated potassium channel family (Rensen et al., 2000). It mainly affects cell adhesion and channel switching while there are no reports that KCNA3 can be methylated in tumors. USP44 is a member of the ubiquitin specific protease family, which regulates mitotic spindle and spindle physical examination (Yang et al., 2019). The results show that USP44 can be directly combined with centrosomal central protein 2 (CETN2) to regulate the spindle geometry, intermediate distance and centrosome separation, while the ubiquitination of CDC20 mediated by USP44 can maintain the integrity of metaphase plate, prevent cell division defects and chromosomal aneuploidy (Stegmeier et al., 2007). In recent years, studies have shown that the expression of USP44 in a variety of tumors has been significantly reduced, including colorectal cancer, breast cancer, esophageal cancer, glioblastoma, renal cancer and testicular cancer (Zhang et al., 2012). In the study of lung cancer, the decrease of USP44 expression level improves the invasive ability of tumor cells and reduces the overall survival rate of patients. The full name of TTC6 gene is Homo sapiens tetratricopeptide repeat domain 6 and its translation product TTC6 protein contains TPR structure and is a TPR protein (Allan & Ratajczak, 2011; Zhang et al., 2019). The important functions of TPR structure include transcriptional control, mitochondrial and peroxisome protein transport, protein kinase inhibition, NADPH oxidase activity, protein folding, immunity and virus replication (D’Andrea & Regan, 2003). The protein structure of TPR structural protein is highly consistent. At the same time, it has many different binding sites, different binding sites play different functions, and functional diversity, the methylation of TTC6 in esophageal squamous cell carcinoma has not been reported. *Smtn* gene, also known as smoothelin gene, encodes the structural protein that constitutes the cytoskeleton. It is specifically expressed in contractile smooth muscle cells, which is related to the contraction of smooth muscle cells and may also be related to the differentiation of smooth muscle cells (Jiang et al., 2012; Engelen et al., 1997). At present, the study of *smtn* gene is still in the exploratory stage, and little is known about its structure and function. Therefore, the methylation of these genes in esophageal squamous cell carcinoma is of great value.

## Conclusion

To sum up, we used a variety of data sets and bioinformatics comprehensive analysis to identify 11,696 pairs of ceRNA relationships in the screening stage. These candidate genes are significantly enriched in multiple pathways, mainly related to FoxO signaling pathway and calcineurin cascade reaction. Through the analysis of TCGA clinical data and the verification of molecular biological methods, four key lncRNAs related to the prognosis of ESCC patients were found, including RNF217-AS1, HCP5, ZFPM2-AS1 and HCG22. These findings can significantly improve our understanding of the etiology and potential molecular events of ESCC, and these candidate lncRNAs and signaling pathways may be used as therapeutic targets of ESCC.

##  Supplemental Information

10.7717/peerj.8831/supp-1 Supplemental Information 1 Differentially expressed miRNAs of GSE59973

10.7717/peerj.8831/supp-2 Supplemental Information 2 Differentially expressed miRNAs of GSE114110

## Additional Information and Declarations

### Data Availability

The following information was supplied regarding data availability: Data is available at NCBI GEO: GSE59973, GSE114110, GSE120356 and GSE52826.
